# Successful use of remimazolam combined with remifentanil for painless gastroscopy in a patient with morbid obesity: a case report

**DOI:** 10.3389/fonc.2024.1383523

**Published:** 2024-07-11

**Authors:** Hai-Shan Feng, Meng-Ran Xie, Yu Meng, Huan-Shuang Pei, Jia-Jia Yu

**Affiliations:** Department of Anesthesiology, The Fourth Hospital of Hebei Medical University, Shijiazhuang, Hebei, China

**Keywords:** remimazolam, remifentanil, morbid obesity, mainless, gastroscopic treatment

## Abstract

**Backgroud:**

In recent years, as the number of people with obesity has surged, the number of morbidly obese patients has also grown. The pathophysiological changes in morbid obesity can lead to combined lung diseases, which may result in hypoventilation, hypoxemia, acute upper airway obstruction, acute respiratory distress syndrome, and sleep apnea syndrome, posing serious challenges to anesthesia management. Here, we describe a case of the administration of remimazolam combined with remifentanil in a patient with morbid obesity undergoing gastroscopy. This has rarely been reported in clinical practice, and we present our management experience here with the aim of providing a reference for clinical work.

**Case presentation:**

We report the case of a 32-year-old male hypertensive patient with a height of 180 cm, weight of 145 kg, and body mass index of 44.8 kg/m^2^. The patient’s main complaint was intermittent hunger pain for more than 1 year, and duodenal polyps were found. Considering the patient’s morbid obesity and the combination of sleep apnea syndrome and hypertension, we administered remimazolam along with remifentanil to ensure perioperative safety.

**Conclusion:**

The procedure lasted 30 min, and the anesthesia was satisfactory with no complications. Remimazolam combined with remifentanil intravenous anesthesia is safe for short gastroscopy in patients with morbidly obesity. The administration of a small dose of split-titration delivery facilitates the maintenance of stable vital signs.

## 1 Introduction

Painless gastroscopy should be performed to minimize patient discomfort and provide appropriate conditions for the endoscopist. As the gastroscope occupies the oral cavity, it increases the difficulty in airway management. Patients with obesity are at increased risk for anesthesia due to a range of physiological and pathological changes in the respiratory and circulatory systems, and obesity has been identified as an independent risk factor for sedation-related adverse events in patients undergoing gastrointestinal endoscopy (1). Owing to their pathophysiological changes, the safety and effectiveness of anesthesia have always been a concern for anesthesiologists. Complications are significantly more common in patients with morbid obesity than in those without, and gastroscopy greatly increases the difficulty of anesthetic management. Fluctuations in anesthesia should be minimized when administered to this group of patients. In this case report, a 32-year-old man with a body mass index (BMI) of 44.8 kg/m^2^ underwent painless gastroscopic duodenal polypectomy. We used remimazolam combined with remifentanil intravenously to safely and effectively complete anesthesia. This report provides new insights into gastroscopic anesthesia in patients with morbid obesity and sleep apnea syndrome.

## 2 Case description

### 2.1 Patient information

A 32-year-old male patient (height, 180 cm; weight, 145 kg; BMI, 44.8 kg/m^2^) complained of duodenal polyps for more than 1 year. The patient was admitted to our hospital because of epigastric pain on hunger with occasional acid reflux, no heartburn, no abdominal distension, and occasional black stools, which appeared more than 1 year earlier without any obvious triggers. Gastroscopy revealed the presence of a descending duodenal polyp. Ball ulcer chronic atrophic gastritis was diagnosed.

The patient had a history of hypertension for more than 1 year, blood pressure (BP) of up to 180/120 mmHg, controlled at 150/110 mmHg, and no history of diabetes mellitus or coronary heart disease. The patient had sleep apnea syndrome that had not been treated. The patient had a history of smoking for more than 10 years (approximately 20 cigarettes per day), no smoking cessation, and occasional low alcohol consumption. There was no family history of hereditary disease.

The patient had a temperature of 36.3°C, a heart rate of 90 bpm, a respiratory rate of 19 breaths/min, and BP of 152/119 mmHg. The patient was conscious, and the physical examination was unremarkable. The respiratory tone in both lungs was slightly lower than normal, and no rales were heard. There was no elevation in the precordial region or rhythm regulation, and no additional heart or pericardial friction sounds were heard.

Routine preoperative laboratory tests revealed no significant abnormalities. Chest computed tomography (CT) showed increased right lower lung texture. Electrocardiography (ECG) revealed sinus arrhythmia. We performed the following assessments: American Society of Anesthesiology class was III, cardiac function grade II, thyromental distance >6 cm, mouth opening > 6 cm, normal neck movement, and Mallampati grade III. The preliminary diagnosis was duodenal polyps, duodenal bulb ulcers, and chronic atrophic gastritis with hypertension.

### 2.2 Anesthetic procedure

**Figure 1:**
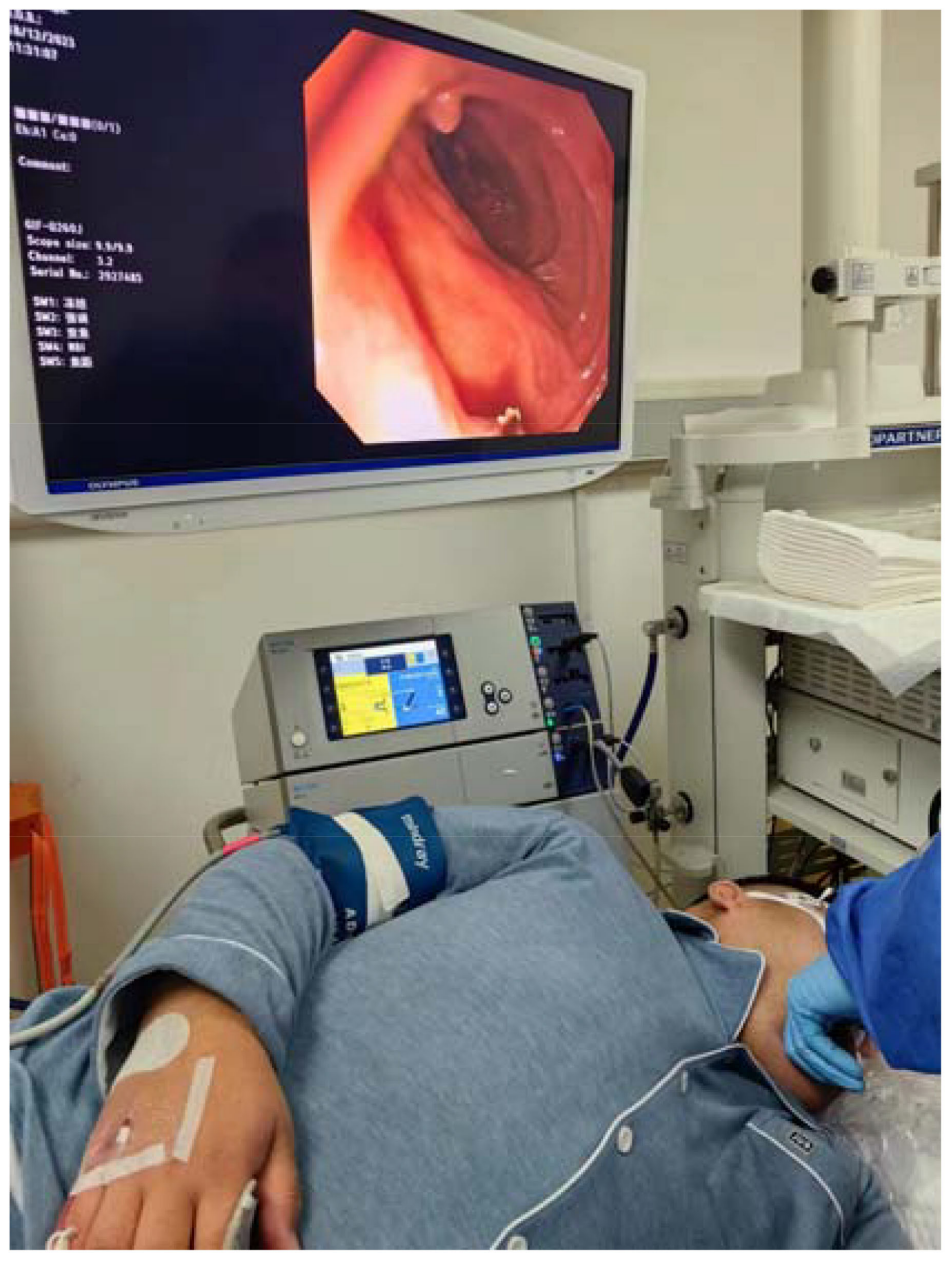
Underwent painless gastroscopic duodenal polypectomy.

**Figure 2:**
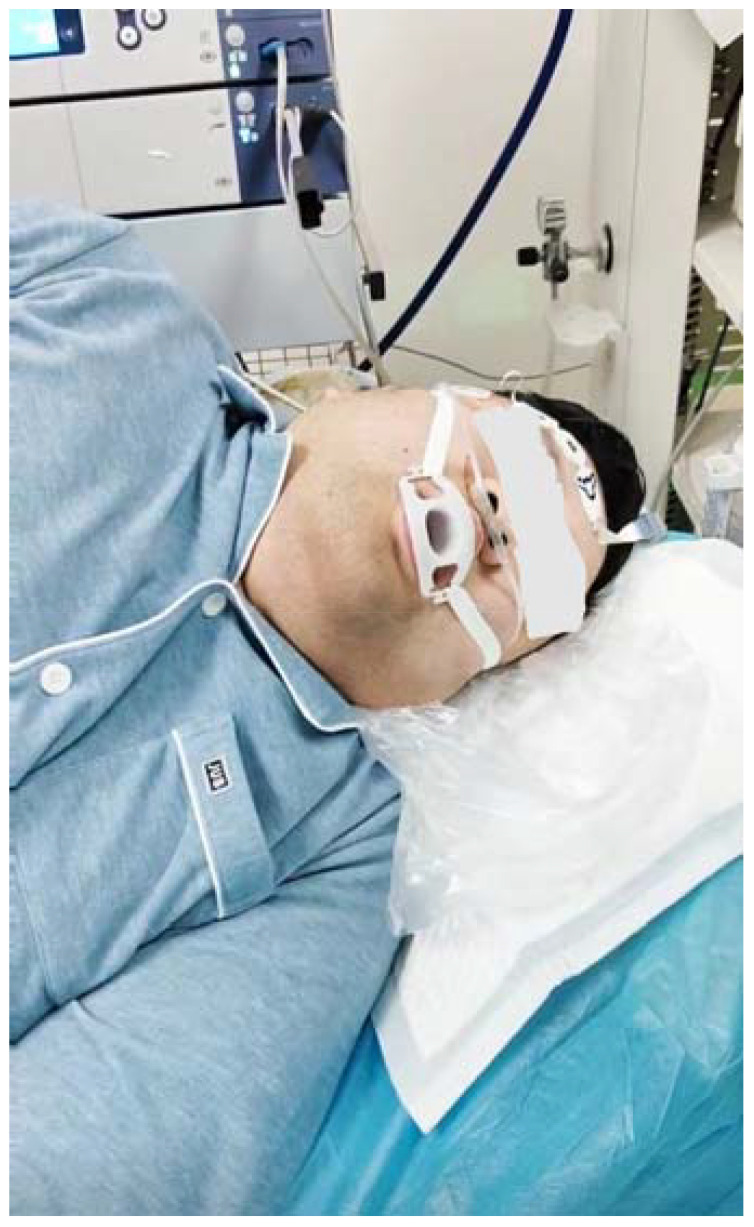
Painless gastroscopic duodenal polypectomy under bispectral index monitoring.

The patient was admitted to the room at 11:10 a.m., and BP, heart rate (HR), pulse oximetry (SpO_2_), respiratory rate (RR), and bispectral index (BIS) were routinely monitored. The following measurements were obtained: BP, 167/89 mmHg; HR, 101 bpm; SpO_2_, 96%; RR, 19 breaths/min, and BIS, 95. The patient laid on his left side and was connected to a nasal cannula with an oxygen intake of 4 L/min. Anesthesia was initiated after complete preoperative preparation. An intravenous shock dose of 36 mg remimazolam (batch number: 231102AU, Jiangsu Hengrui Medicine Co., Lianyungang, China) (0.25 mg/kg) and 15 μg remifentanil (batch number: 30A09152, Yichang Renfu Pharmaceutical Co., Yichang, China) (0.1 μg/kg) was injected. When the patient’s respiration slowed down, the eyelash reflex disappeared, and jaw relaxation started, gastroscopy began, and at this time, the BIS was maintained between 65 and 70. According to the patient’s physical signs, such as deepening respiration, increasing HR, and body movement, single additional doses of 5 mg remimazolam and 10 μg remifentanil were administered. When the patient presented with SpO_2_ ≤ 92%, he was given open-airway treatment by supporting the lower jaw; when SpO_2_ ≤ 90%, he was given oxygen by face-mask. This treatment lasted 30 min, and BP was maintained at 150–160/90–96 mmHg, HR was 80–91 bpm, SpO_2_ was maintained at 92%–99%, RR was 12–16 breaths/min, and BIS was 65–70 during treatment. There was no body movement or emergency airway during the operation, but there were 2 times when SpO_2_ dropped to 92%. To avoid further drop of SpO_2_, immediate treatment of opening the airway by supporting the lower jaw was given, and SpO_2_ was improved. Intraoperative vital signs were stable and the treatment was completed successfully. The total dosages of remimazolam and remifentanil were 70 mg and 100 μg, respectively. At the end of treatment, the patient’s vital signs were as follows: BP, 158/89 mmHg; HR, 80 bpm; SpO_2_, 99%; RR, 15 breaths/min; BIS, 68. At the end of the operation, the patient was injected with 0.5 mg flumazenil intravenously, and after 1 min, the patient woke up with the following vital signs: BP, 157/80 mmHg; HR, 73 bpm; SpO_2_, 99%; RR, 16 breaths/min; BIS, 96 (**Figures 1**, **2**). Upon awakening, the patient reported a good sleep, no memory of the treatment process, no discomfort such as chest tightness, shortness of breath, pain, or nausea, and was very satisfied with this painless gastroscopic treatment. After 20 min of observation, the patient’s vital signs were stable, with no complaints of discomfort, and a Steward score of 6, and he returned to the ward peacefully.

### 2.3 Follow-up and outcomes

Twenty-four hours later, the patient did not complain of chest tightness, shortness of breath, dizziness, nausea, or other issues. He recovered and was discharged from the hospital 3 days after surgery without respiratory complications. The postoperative diagnosis was duodenal polyps, duodenal bulb ulcers, and chronic atrophic gastritis with hypertension. Two months after surgery, the patient did not complain of discomfort at a telephone follow-up (**Table 1**).

**Table 1:** Case report timeline.

Item		Timeline
Preoperative	1	Duodenal polyps for more than >1 year were discovered.
	2	The patient had a history of hypertension for more than 1 year, blood pressure up to 180/120 mmHg, controlled at 150/110 mmHg, and denied a history of diabetes mellitus or coronary heart disease. The patient had sleep apnea syndrome, which had not been treated.
	3	Chest CT: increased right lower lung texture.
	4	The patient required a painless gastroscopic duodenal polypectomy.
**Perioperative**	5	The patient was admitted to the room at 11:10 a.m., and BP, HR, SpO_2_, RR, and BIS were routinely monitored.
	6	The patient was lying on the left side and was connected to a nasal cannula for oxygen intake of 4 L/min.
	7	To prevent the emergence of an emergency airway during the operation, the following airway tools were prepared before the anesthesia: mask, oropharyngeal ventilation, laryngeal mask, and tracheal tube.
	8	Vasoactive drugs: norepinephrine, uradil hydrochloride, atropine, and other medicines were also prepared in case of emergency.
	9	Anesthesia was started. An intravenous shock dose of remimazolam 36 mg (0.25 mg/kg) and remifentanil 15 ug (0.1 μg/kg) was injected.
	10	According to the patient’s physical signs, such as deepening respiration, increasing HR, and body movement, a single additional dose of 5 mg remimazolam and 10 μg remifentanil was administered.
	11	When the patient’s respiration slowed, the eyelash reflex disappeared, and jaw relaxation started.
	12	There was no body movement or emergency airway during the operation, but there were 2 times when SpO_2_ dropped to 92%. To avoid further drop of SpO_2_, immediate treatment of opening the airway by supporting the lower jaw was given, and the SpO_2_ was improved.
**Postoperative**	13	Upon awakening, the patient reported a good sleep, no memory of the treatment process, no discomfort such as chest tightness, shortness of breath, pain, or nausea, and was very satisfied with this painless gastroscopic treatment.
	14	After 20 min of observation, the patient’s vital signs were stable, with no complaints of discomfort and a Steward score of 6, and returned to the ward peacefully.
	15	The patient was discharged 3 days after treatment.
	16	Two months after treatment, the patient did not complain of discomfort on telephone follow-up.

ECG, electrocardiography; SpO_2,_ pulse oximetry; BIS, bispectral index; CT, computed tomography; BP, blood pressure; HR, heart rate; RR, respiratory rate.

## 3 Discussion

Herein, we report the case of a 32-year-old male with a BMI of 44.8 kg/m^2^ who was to undergo gastroscopic duodenal polypectomy. We used remimazolam combined with remifentanil intravenously to safely and effectively complete anesthesia.

As people have become more aware of the need for medical checkups in recent years, the number of painless gastroscopies and treatments has increased. The number of special populations encountered is also increasing, as are the challenges faced by anesthesiologists. With improvements in living standards, the number of people with obesity is increasing and they are presenting younger. Studies have shown that patients with obesity are significantly more likely than patients with healthy weight to experience hypoxemia and respiratory interventions during gastroscopy visits in a sedated state (2). The BMI of this patient was as high as 44.8 kg/m^2^, which is categorized as morbidly obese, and he was facing proposed treatment of gastroscopic polypectomy, which was a serious challenge for anesthesia.

BMI is currently the most widely used measure for evaluating obesity. According to the diagnostic criteria of the World Health Organization (WHO) (3), morbid obesity is defined as BMI ≥35 kg/m^2^. Patients with morbid obesity have different degrees of abnormal fat distribution and accumulation in tissues throughout the body, which results in a large amount of soft-tissue hyperplasia in the oropharynx, muscle relaxation, sagging, and hyperplasia of the tongue, leading to a relative reduction in the volume of the oropharyngeal cavity and a relative reduction in the upper part of the tongue, leading to a decrease in the volume of the oropharyngeal cavity and a narrower upper airway. As a result, patients with morbid obesity tend to have comorbid obstructive sleep apnea syndrome and a high risk of difficult airway (4). Excessive thoracic and abdominal fat leads to thoracic and lumbar lordosis, limiting intercostal muscle movement and thoracic expansion. Abdominal distension reduces respiratory muscle function and leads to reduced compliance of the chest wall and lungs, resulting in a 35% reduction in total respiratory compliance (5). Although the present patient’s mouth opening and chin–nail distance was normal (Mallampati III), the risk of anesthesia increased owing to excessive fat accumulation in the neck and pharynx, which can narrow the airway lumen after the induction of anesthesia, making the patient highly susceptible to fatal acute upper airway obstruction. Gastroscopy is the main use of anesthesia outside the operating room. During painless gastroscopy and treatment, the digestive endoscope occupies the patient’s oral cavity and interferes with respiratory management, greatly increasing the risk of anesthesia and respiratory management difficulty. The choice of anesthesia regimen requires extra attention; anesthesia should not be too deep, causing an increase in the risk of respiratory depression, and it should not be too shallow, resulting in patient movement, which can interfere with the procedure. Therefore, accurate sedation and analgesia need to be provided. The proposed procedure for this patient was endoscopic duodenal polypectomy, with an estimated operative time of approximately 20–30 min.

Both general and intravenous anesthesia can be administered. Each type of anesthetic has its advantages and disadvantages. General anesthesia not only ensures the patient’s airway patency and adequate oxygen supply but also provides safe and effective surgical conditions for the operator, especially avoiding agitation caused by the patient’s body movements. However, owing to increased intra-abdominal pressure and diaphragmatic elevation caused by the accumulation of abdominal fat, lung infection can easily develop after general anesthesia. Some studies have shown (6) that patients with morbid obesity have a certain area of pulmonary atelectasis before the induction of anesthesia; the location of pulmonary atelectasis increases rapidly after mechanical ventilation and pulmonary atelectasis is still not significantly reduced 24 h after surgery. Obesity affects the distribution, protein binding, and excretion of anesthetic drugs, and the accumulation of anesthetic drugs in adipose tissue causes secondary redistribution. Owing to the short duration of surgery, the drugs are not metabolized completely after general anesthesia, which can easily cause delayed awakening and affect the surgical turnover rate.

Intravenous anesthesia is commonly used in painless gastroscopy, providing a safe and comfortable environment. However, if it is administered for a long time, it may lead to carbon dioxide accumulation and hypoxia. Delayed awakening due to prolonged intravenous drug use leads to drug accumulation. In this case, the operative time was expected to be approximately 30 min; therefore, selecting appropriate intravenous drugs is particularly important.

Propofol is commonly used for painless gastroscopy; however, there are some limitations, such as the tendency for respiratory depression, hypotension, and injection pain (7), and respiratory and circulatory inhibition increases with an increase in the dose. Prolonged use can cause drug accumulation in the absence of a specific antagonist, resulting in delayed awakening. Cypropofol is a new type of intravenous sedative anesthetic drug that, when applied for a long period, can cause adverse effects such as hypotension, respiratory depression, drug accumulation, and delayed awakening. Remimazolam is a new type of benzodiazepine sedative that is rapidly hydrolyzed into an inactive metabolite by enzymes in the body. It has the characteristics of rapid onset of action, rapid awakening, and less interference with circulation. In recent years, it has been approved for use in general anesthesia in Japan, the United States, and Europe. Several studies have shown that remimazolam significantly reduces the incidence of adverse events such as respiratory depression, hypotension, and bradycardia compared with propofol (8) and has the advantage of being a specific antagonist. Remimazolam was administered at 0.1–0.3 mg/kg in the present patient. The onset of action was 1–3 min, with a peak time of approximately 1 min. In the present case, the shock dose was 36 mg (0.25 mg/kg), and 5 mg of additional drug was administered per time based on the observation of changes in the patient’s physical signs, such as deepening of respiration, increase in HR, and bradycardia. The pharmacokinetics of remimazolam are linear, with no significant correlation between clearance and bodyweight, and there is a low likelihood of accumulation and prolongation of effects with a prolonged infusion or high dosage of the drug (9), which makes it a better choice for patients with morbid obesity. In this case, the patient received 70 mg of intravenous remimazolam, but the success rate of sedation with remimazolam alone was low, and there were many adverse reactions, such as eructation and choking. The use of opioids in conjunction with remimazolam can address these shortcomings.

Nalbuphine, oxycodone, fentanyl, sufentanil, and remifentanil are commonly administered in clinical practice. Nalbuphine is an opioid receptor agonist–antagonist with comparable analgesic effects to morphine, with low adverse effects such as respiratory depression, nausea, and vomiting; however, its half-life and action time are long, especially for patients with morbid obesity. This can easily cause accumulation and secondary distribution of the drug, which is not conducive to patient awakening and safety. Oxycodone is a κ,μ double-opioid receptor agonist, with good analgesia and light inhibition of circulatory respiration. However, its action time is long, and drug accumulation is easy, which is not a good choice for patients with morbid obesity. Fentanyl causes respiratory depression, asphyxia, and chest wall muscle rigidity (10). Fentanyl’s time–volume half-life increases with infusion time; after stopping the drug, the blood concentration decreases slowly, and the drug accumulates in the fat, which can cause an increase in intragastric pressure, and nausea and vomiting are common postoperative side effects. Sufentanil can easily cause dizziness, drowsiness, nausea, and vomiting and is metabolized by the liver; it has a long half-life and is not conducive to early recovery. As a short-acting opioid receptor agonist, remifentanil can be rapidly hydrolyzed to inactive metabolites by specific esterases in the plasma and tissues, with a fast onset of action and strong analgesic effect, and can effectively inhibit choking during entry. The continuous infusion half-life is short, metabolism is not affected by liver and kidney function, there is no accumulation effect, and there is less residual effect of postoperative anesthesia, which can ensure the safety of patients after leaving the hospital. Patients with obesity should preferably use drugs with faster metabolism, no accumulation, and less impact on the respiratory system. Patients with obesity should avoid the use of long-acting opioids to prevent nausea and vomiting. Autonomous breathing resumed quickly after remifentanil was discontinued in this patient, and there were no nausea or vomiting side effects. We administered 15 μg (0.1 μg/min) of remifentanil intravenously 1 min before the operator introduced the gastroscope, and this dose was based on previous experience with painless gastroscopy.

Intravenous general anesthesia in patients with obesity and those who snore should be performed with attention to the balance between patient comfort and anesthesia safety. Propofol is currently a commonly used anesthetic drug in painless gastroscopy, with the advantages of fast onset, rapid recovery, and high clearance, but it has a strong inhibitory effect on the cardiovascular and respiratory systems and is dose-dependent with a high incidence of side effects, which are particularly evident in elderly adults and patients with obesity (11). Borkett et al. (12) reported that during endoscopy, the sedation effect of remimazolam alone was exact, but the patients had more somatic reactions and low satisfaction with the examination. To reduce the occurrence of somatic reaction, an appropriate amount of analgesic such as remifentanil can be given in advance, and a balance between patient comfort and safety can be achieved by compounding the medication.

Remimazolam compounded with remifentanil has a synergistic effect (13): the dosage of the compound application is significantly lower than when either are used alone, the inhibitory effect on the patient’s circulatory and respiratory systems is significantly weakened, and a more satisfactory anesthesia effect can be obtained. The reason for the high success rate of first-dose sedation with remimazolam compounded with opioids may be that the binding of opioids to opioid receptors in the medulla oblongata inhibits the occurrence of choking. It has been shown that remimazolam combined with remifentanil for gastroscopy can improve the sedation rate, reduce adverse effects, and shorten the hospitalization time (14). The rational application of remimazolam and remifentanil can effectively and safely control the depth of anesthesia and reduce postoperative side effects. In this case, we used an anesthesia protocol of remimazolam combined with remifentanil intravenously. To ensure safety, we optimized the drug delivery method, using a small dose of split-titration delivery (remimazolam 5 mg/dose, remifentanil 10 μg/dose). This protocol can effectively maintain the depth of anesthesia and reduce fluctuations in anesthesia while avoiding the excessive use of drugs. Although this treatment was completed safely and effectively, there was a certain degree of anesthesia risk, and so it was critical to have adequate anesthesia planning and preparation just in case. To prevent the emergence of an emergency airway during the treatment, a mask, oropharyngeal ventilation, laryngeal mask, and tracheal tube were prepared before anesthesia. Vasoactive drugs, such as norepinephrine, uradil hydrochloride, atropine, and other medicines, were also prepared in case of an emergency. In addition, general anesthesia by tracheal intubation is still a more appropriate choice for patients with obesity who are expected to have a large number of polyps, which makes gastroscopy more difficult and prolongs the treatment time. If bleeding or other complications occur during treatment of patients with obesity under intravenous anesthesia for gastroscopic resection of a single polyp or other short surgical procedures, it is necessary to make preparations to change to general anesthesia with endotracheal intubation to ensure patient safety. More attention should be paid for patients with morbid obesity.

In this report, we describe the successful use of intravenous anesthesia with remimazolam combined with remifentanil in a patient with morbid obesity under BIS monitoring for a short gastroscopy. However, our findings need to be confirmed in future clinical trials. This case report aimed to provide new ideas for the use of clinical anesthesia in such patients.

As the number of people with obesity has surged, the number of morbidly obese patients has also grown. Owing to their pathophysiological changes, the safety and effectiveness of anesthesia have always been a concern for anesthesiologists. We used remimazolam combined with remifentanil intravenously to safely and effectively complete anesthesia. The administration of a small dose of split-titration delivery facilitates the maintenance of stable vital signs. This report provides new insights into gastroscopic anesthesia in patients with morbid obesity and sleep apnea syndrome.

## Data availability statement

The original contributions presented in the study are included in the article/supplementary material. Further inquiries can be directed to the corresponding author.

## Ethics statement

Written informed consent was obtained from the individual(s) for the publication of any potentially identifiable images or data included in this article.

## Author contributions

H-SF: Writing – original draft. M-RX: Investigation, Writing – review & editing. YM: Investigation, Writing – review & editing. H-SP: Validation, Writing – original draft, Writing – review & editing. J-JY: Investigation, Writing – review & editing.

## References

[1] Laffin AE Kendale SM Huncke TK. Severity and duration of hypoxemia during outpatient endoscopy in obese patients: a retrospective cohort study. Can J Anaesth. (2020) 67:1182–9. doi: 10.1007/s12630-020-01737-x 32514693

[2] Wani S Azar R Hovis CE Hovis RM Cote GA Hall M. Obesity as a risk factor for sedation-related complications during propofol-mediated sedation for advanced endoscopic procedures. Gastrointest Endosc. (2011) 74:1238–47. doi: 10.1016/j.gie.2011.09.006 PMC4124898 22136773

[3] Obesity WHOCo. Preventing and managing the global epidemic. Geneva: World Health Organization (2000). World Health Organization Obesity. 11234459

[4] Lin CC Liu KH Lee LA Chuang LP Lin YS Hsin LJ. Combined Airway and Bariatric Surgery (CABS) for obstructive sleep apnea in obese patients with Morbid Obesity: A comprehensive alternative preliminary study. J Clin Med. (2022) 11:7078. doi: 10.3390/jcm11237078 36498653 PMC9738588

[5] Cai XC Zhou ZH Zhu W. Meta-analysis of the effects of different levels of positive end-expiratory pressure on intraoperative oxygen and index in obese patients. J Clin Anesthesiol. (2022) 38:507–12. doi: 10.12089/jca.2022.05.012

[6] Sprung J Whalley DG Falcone T Wilks W Navratil JE Bourke DL. Effects of the tidal volume and respiratory rate on oxygenation and respiratory mechanics during laparoscopy in morbidly obese patients. Anesth Analg. (2003) 97:268–74. doi: 10.1213/01.ANE.0000067409.33495.1F 12818980

[7] Bushuven S Heise D. Propofol up2date - Teil 2: Patientengruppen, unerwünschte Wirkungen und die Nachfolgesubstanz Fospropofol [Propofol up2date]. Anesthesiol-Intensivmed Notfallmed Schmerzther. (2013) 48:444–51. doi: 10.1055/s-0033-1352489 23929162

[8] Hu B Jiang K Shi W Xiao S Zhang S Tan C. Effect of remimazolam tosilate on respiratory depression in elderly patients undergoing gastroscopy: A multicenter, prospective, and randomized study. Drug Des Devel Ther. (2022) 16:4151–9. doi: 10.2147/DDDT.S391147 PMC9733689 36506792

[9] Ma GS Chen XH Cao HZ. Application of remimazolam for painless gastroscopy in elderly obese patients. J Clin Anesthesiol. (2022) 38:1057–60. doi: 10.12089/jca.2022.10.009

[10] Yin N Xia J Cao YZ Lu X Yuan J Xie J. Effect of propofol combined with opioids on cough reflex suppression in gastroscopy: study protocol for a double-blind randomized controlled trial. BMJ Open. (2017) 7:e014881. doi: 10.1136/bmjopen-2016-014881 PMC5589021 28864688

[11] Wang HX Qiu HR Wei W Jin M Tian M Xue FS. A comparison of sevoflurane-propofol versus sevoflurane for insertion of BlockBuster device in obese patients undergoing bariatric surgery: a randomized, double-blinded controlled study. Int J Anesthesiology Resuscitation. (2020) 41:558–62. doi: 10.3760/cma.j.cn321761-20190911-00051

[12] Borkett KM Riff DS Schwartz HI Winkle PJ Pambianco DJ Lees JP. A Phase IIa, randomized, double-blind study of remimazolam (CNS 7056) versus midazolam for sedation in upper gastrointestinal endoscopy. Anesth Analg. (2015) 120:771–80. doi: 10.1213/a.0000000000000548 25502841

[13] Kops MS Pesic M Petersen KU Schmalix WA Stöhr T. Impact of concurrent remifentanil on the sedative effects of remimazolam, midazolam and propofol in cynomolgus monkeys. Eur J Pharmacol. (2021) 890:173639. doi: 10.1016/j.ejphar.2020.173639 33065095

[14] Peng R Zhang JY Wang Q Yang TS Liu HQ Sun JH. Effect of remimazolam combined with different opioids in painless gastroscopy. Int J Anesthesiology Resuscitation. (2023) 44:838–42. doi: 10.3760/cma.j.cn321761-20230329-00860

